# Supplementary material for: Single Cell RNA Sequencing Provides Clues for the Developmental Genetic Basis of Syngnathidae’s Evolutionary Adaptations
Source: bioRxiv. 2024 Apr 9:2024.04.08.588518. Preprint. [Version 1] doi: 10.1101/2024.04.08.588518 (PMC11030337; doi:10.1101/2024.04.08.588518)
Supplement: Supplementary file 1 [file NIHPP2024.04.08.588518v1-supplement-1.pdf]

bioRxiv preprint doi: https://doi.org/10.1101/2024.04.08.588518; this version posted April 9, 2024. The copyright holder for this preprint (which was not certified by peer review) is the author/funder, who has granted bioRxiv a license to display the preprint in perpetuity. It is made available under aCC-BY 4.0 International license.

Supplementary file captions:

SupplementaryFile1: 230816_scRNAseq_cellranger_info.csv, a table containing quality metrics for the single cell libraries.

SupplementaryFile2: 230818_Cell_Cluster_Sizes. csv, a table containing the number of cells in each cell cluster and cluster identities.

SupplementaryFile3: Selected_Markers_Information.xlsx, a file containing additional information on the marker gene identified for every cluster.

SupplementaryFile4: In_Situ_Probe_and_Samples_Used_SuccessfulProbes.xlsx, a list of the in situ probes used in this study, the conditions used to prepare the probes, and the staging/sample information for the embryos.

bioRxiv preprint doi: https://doi.org/10.1101/2024.04.08.588518; this version posted April 9, 2024. The copyright holder for this preprint (which was not certified by peer review) is the author/funder, who has granted bioRxiv a license to display the preprint in perpetuity. It is made available under aCC-BY 4.0 International license.

SupplementaryFile5: WGCNA_networks_with_descriptions.xlsx, a file containing all the genetic networks, the genes inside of them, and additional information for the genetic networks highlighted in this paper.

SupplementaryFile6: WGCNA_module_number_to_color_translation_table, genetic networks were initially labeled with colors, we converted these labels to numeric annotations for simplicity using this conversion table. The table also contains the number of genes in each network.

SupplementaryFile7: 240228_ssc_WGCNA_mod_association_wClus_tstat.csv, this file contains the t-statistics derived for each module-cell cluster pair. The cell clusters are in the rows and the gene modules are in the columns.

SupplementaryFile8: 240228_ssc_WGCNA_associat_mods_w_clus_tstat_pvalues_corrected.csv, p-values for the t-statistics of the strength of association between gene modules and cell clusters. P-values are corrected for multiple testing hypotheses using fdr. The cell clusters are in the rows and the gene modules are in the columns.

SupplementaryFile9: 240227_WGCNA_connectivity_change.csv, the change in connectivity for gene modules when individual cell clusters are removed. The cell clusters are in the columns and the gene networks are in the rows.

SupplementaryFile10: 240228_WGCNA_EM1_EM2_Connectivity_Permutation_Pvalues_Corrected.csv, the p-values for the change in connectivity are found in this csv. The cell clusters are in the columns and the gene networks are in rows.

bioRxiv preprint doi: https://doi.org/10.1101/2024.04.08.588518; this version posted April 9, 2024. The copyright holder for this preprint (which was not certified by peer review) is the author/funder, who has granted bioRxiv a license to display the preprint in perpetuity. It is made available under aCC-BY 4.0 International license.

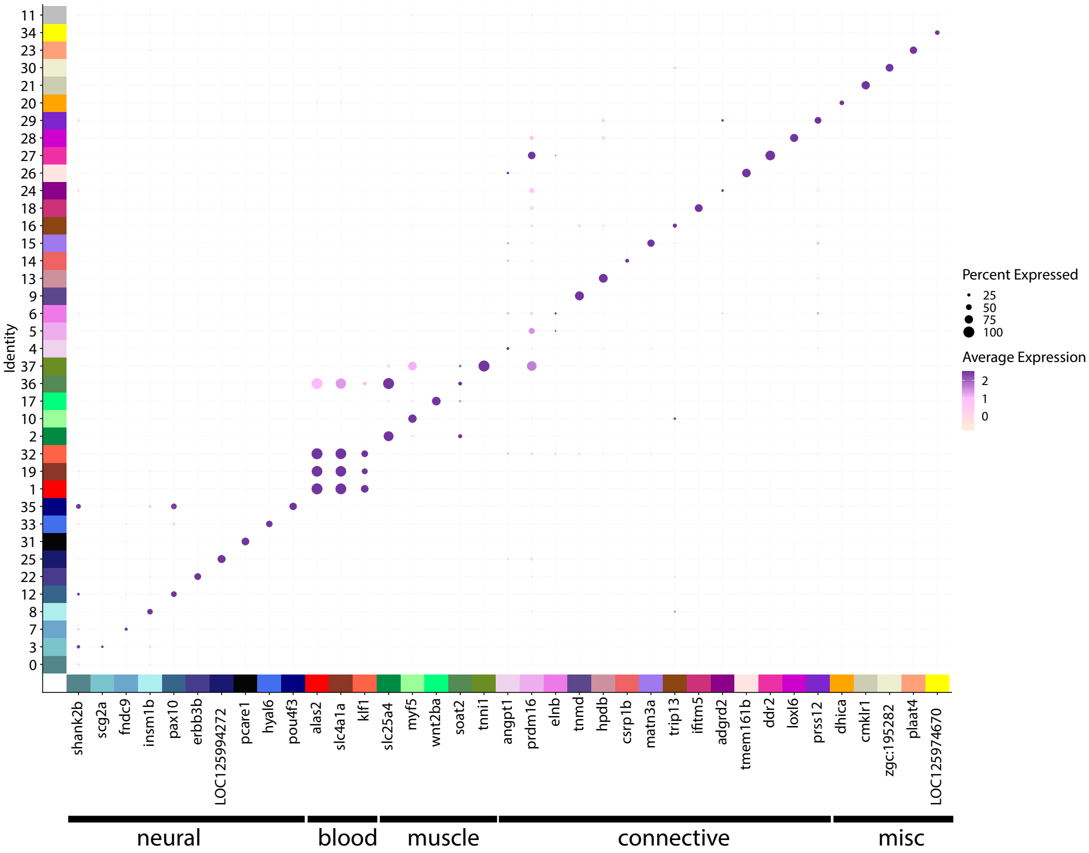

Figure S1: *Marker genes define cell identity and are a resource for cell cluster exploration.* This dotplot shows the marker genes on the x-axis, and the cell cluster on the y-axis. The size of each dot indicates the percentage of cells that express the gene in each cluster. The darkness of each dot represents the average expression of the gene.

bioRxiv preprint doi: https://doi.org/10.1101/2024.04.08.588518; this version posted April 9, 2024. The copyright holder for this preprint (which was not certified by peer review) is the author/funder, who has granted bioRxiv a license to display the preprint in perpetuity. It is made available under aCC-BY 4.0 International license.

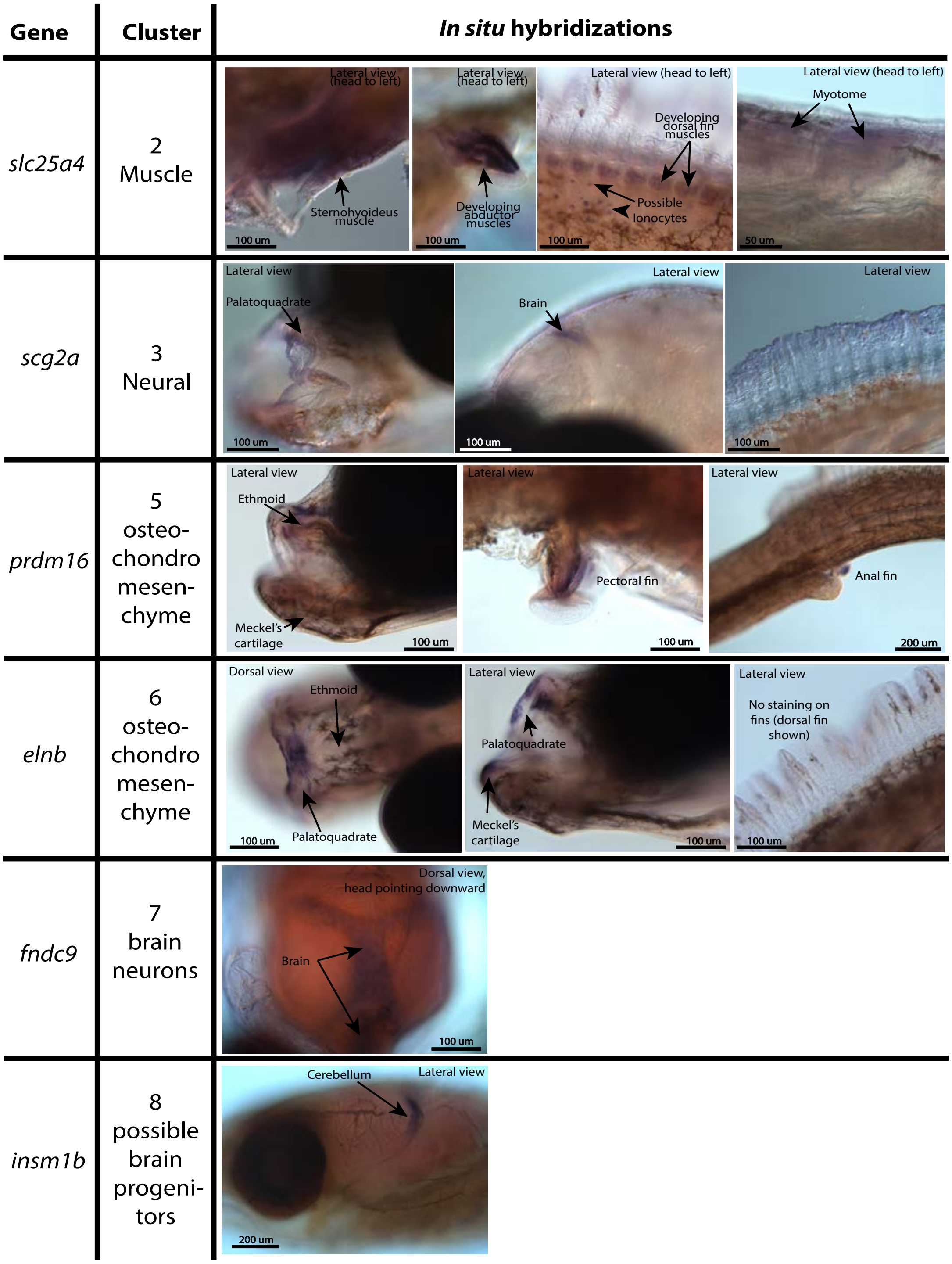

Figure S2: *Marker genes examined through in situ hybridization reveal spatial patterning of cell types.*Informative marker gene *in situ* hybridization results, including *slc25a4*, *scg2a*, *prdm16*, *elnb*, *fndc9*, and *insm1b*. Embryos are staged to highlight staining of each gene. Relavant anatomical features are distinguished with arrows. One day post spawn Gulf pipefish larvae were used for *fndc9 in situs*, five day post spawn Gulf pipefish larvae were used for *insm1b in situs*, and wild caught bay pipefish at the onset of craniofacial elongation were used for all other probes.

bioRxiv preprint doi: https://doi.org/10.1101/2024.04.08.588518; this version posted April 9, 2024. The copyright holder for this preprint (which was not certified by peer review) is the author/funder, who has granted bioRxiv a license to display the preprint in perpetuity. It is made available under aCC-BY 4.0 International license.

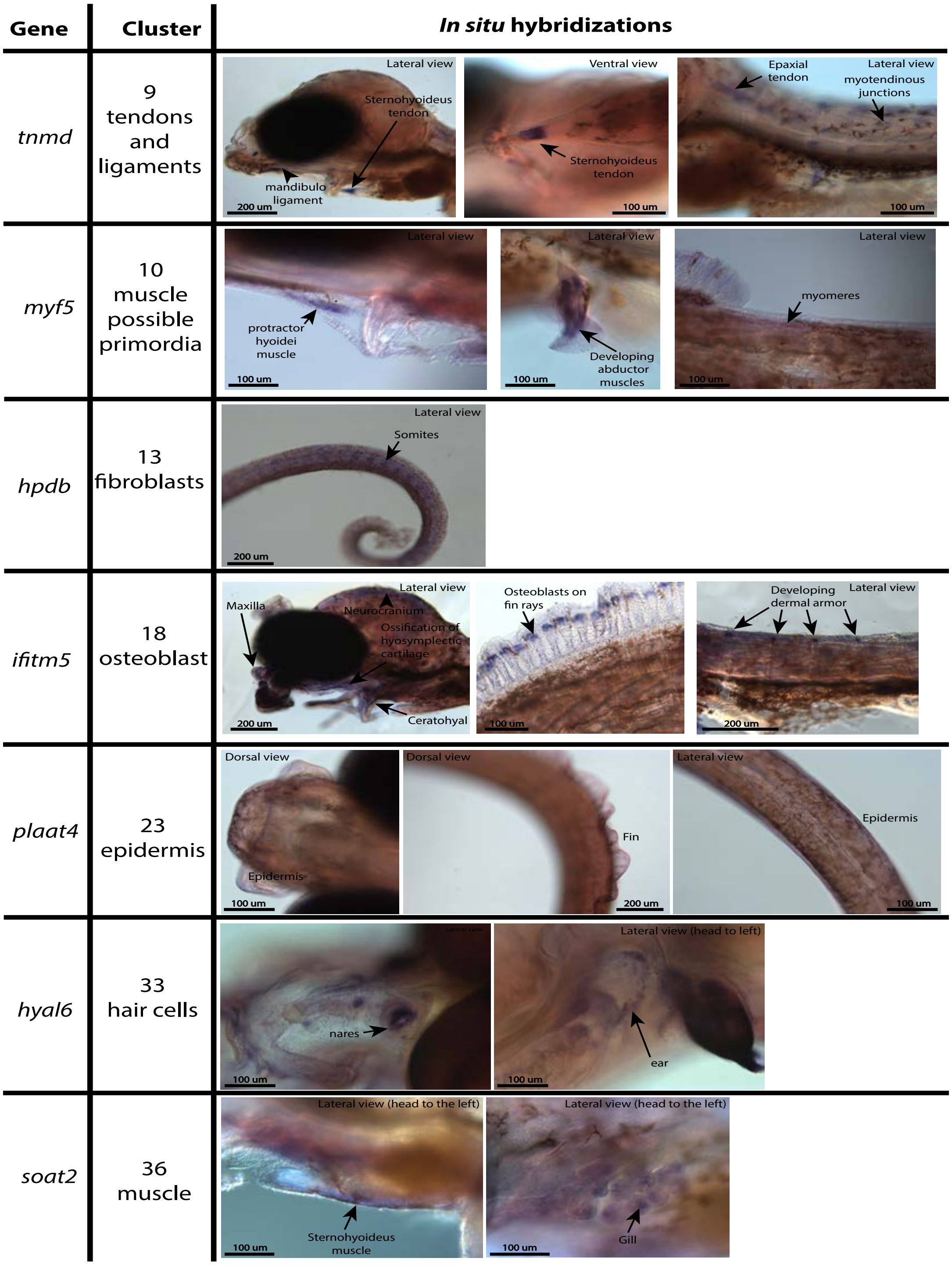

Figure S3: *Marker genes examined through in situ hybridization reveal spatial patterning of cell types.* Informative marker gene *in situ* hybridization results, including *tnmd*, *myf5*, *hpbd*, *ifitm5*, *plaat4*, *hyal6*, and *soat2*. Embryos are staged to highlight staining of each gene. Relavant anatomical features are distinguished with arrows. All *in situs* were completed with wild caught bay pipefish: one day post spawn larvae were used for the *hyal6* probe, mid-somitogenesis embryos were used for the *hpbd*, and embryos at the onset of craniofacial elongation were used for all other probes.

bioRxiv preprint doi: https://doi.org/10.1101/2024.04.08.588518; this version posted April 9, 2024. The copyright holder for this preprint (which was not certified by peer review) is the author/funder, who has granted bioRxiv a license to display the preprint in perpetuity. It is made available under aCC-BY 4.0 International license.

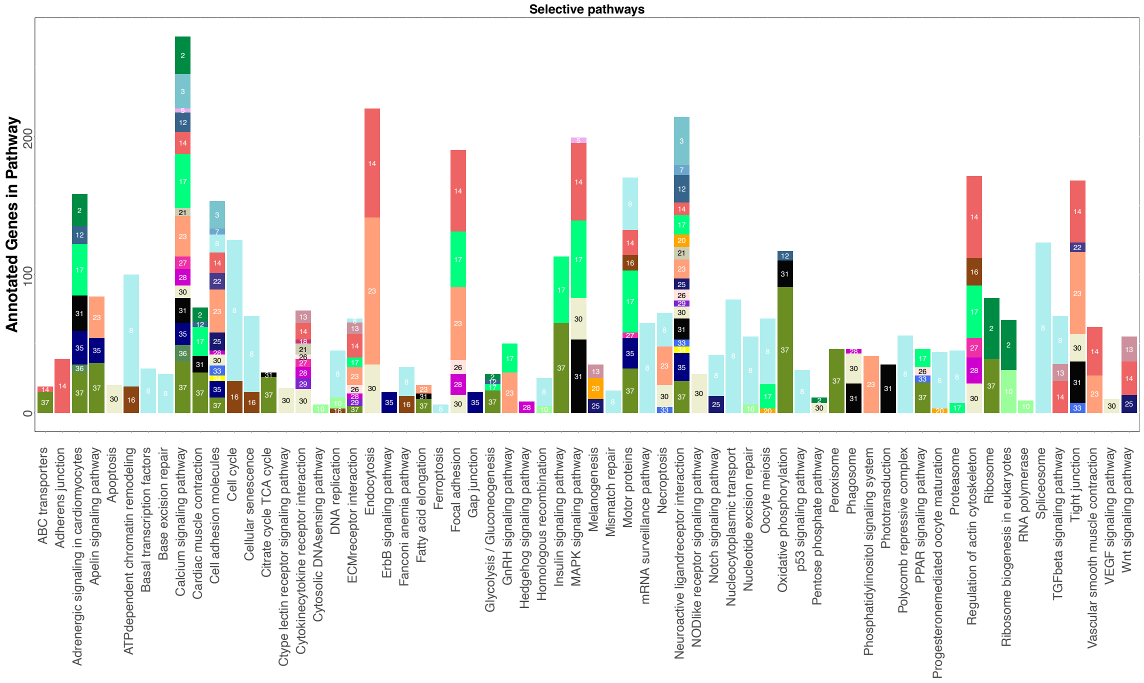

Figure S4: *KEGG analysis reveals pathways signficiantly enriched in cell clusters.* KEGG pathways are listed on the x-axis. For each cluster, the number of genes that belong to the specific KEGG pathway is represented by the height of each bar. Cell clusters are labeled by both color and their designated number. For clarity, only cell clusters with more than three annotated genes in each pathway are shown and certain pathways involving infection and metabolism are removed.

bioRxiv preprint doi: https://doi.org/10.1101/2024.04.08.588518; this version posted April 9, 2024. The copyright holder for this preprint (which was not certified by peer review) is the author/funder, who has granted bioRxiv a license to display the preprint in perpetuity. It is made available under aCC-BY 4.0 International license.

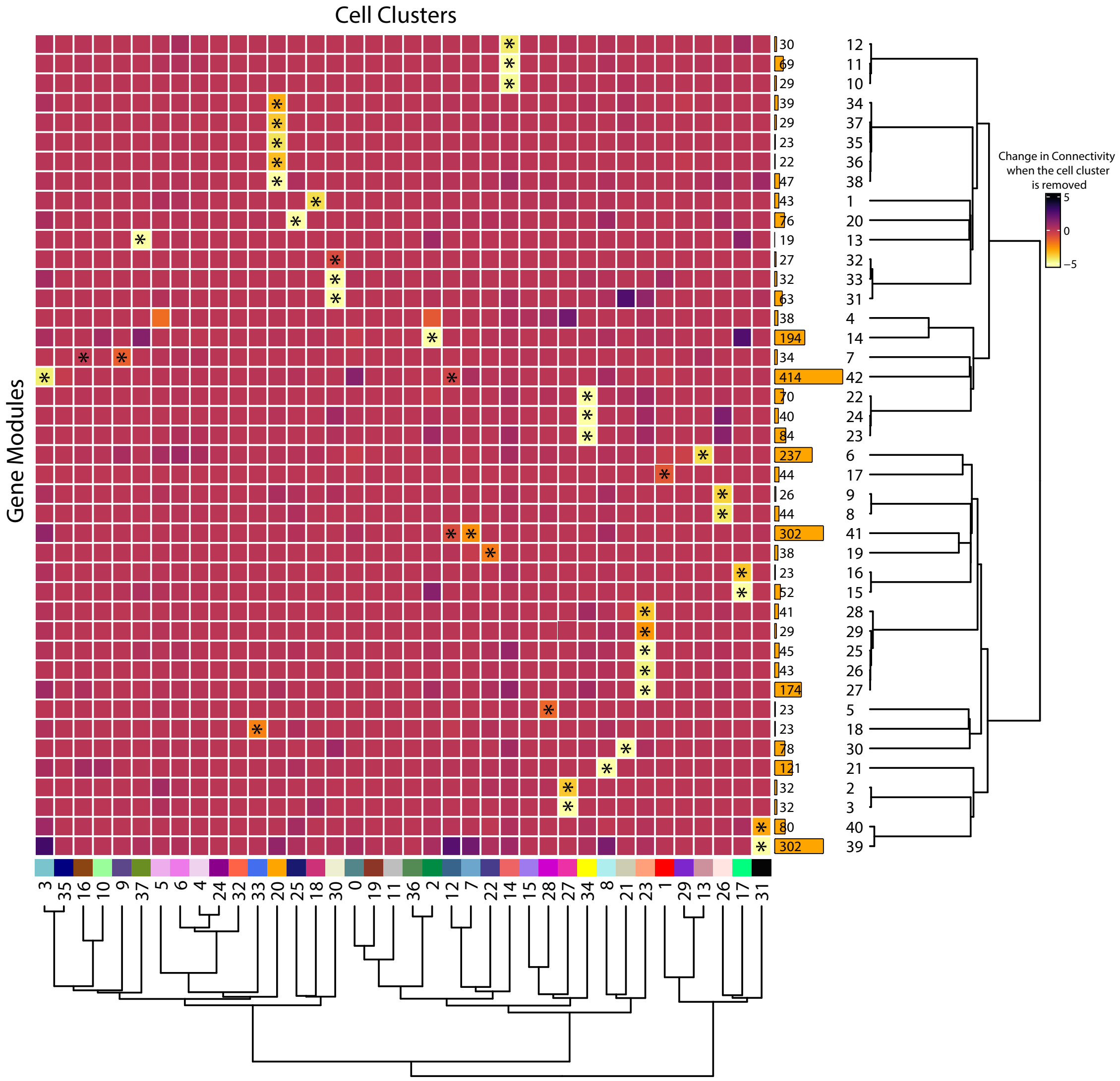

Figure S5: *Cell clusters drive gene module connectivity.* The heatmap shows the change in gene network connectivity when individual cell clusters are removed. Cell clusters are in the columns and gene modules are in rows. The coloration scale is based on the change in connecitvity when a cell cluster is removed, with yellow indicating a large change in connectivity. The modules and clusters are clustered using the Pearson distance method. Asterisks indicate cluster-module pairs that are significant using a one way permutation test and FDR correction. The size of the gene modules are shown to the right.

bioRxiv preprint doi: https://doi.org/10.1101/2024.04.08.588518; this version posted April 9, 2024. The copyright holder for this preprint (which was not certified by peer review) is the author/funder, who has granted bioRxiv a license to display the preprint in perpetuity. It is made available under aCC-BY 4.0 International license.

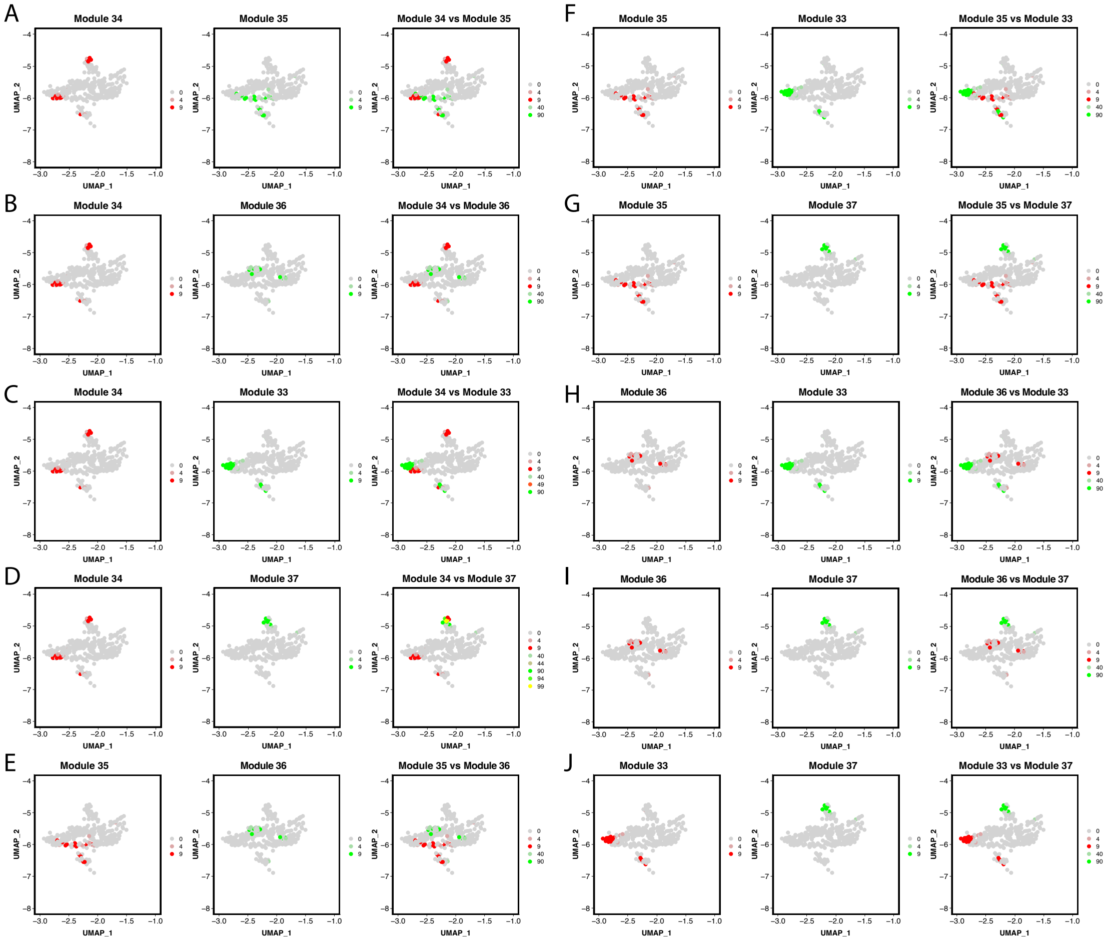

Figure S6: *Gene module expression comparison with the Pigment Cluster reveals potential elusive cell types within the cluster.* Genes within the network are combined into one feature then plotted on a feature plot. In this case, UMAP dimensions one and two are the x and y-axis accordingly, dots represent each pigment cell, and the color of the dots represents the expression of the module. Cells that express gene modules are either colored red or green (when modules are plotted on separate plots). When plotted on the same plot, cells that express both gene modules are yellow. Panels A through J show each gene module comparison.

bioRxiv preprint doi: https://doi.org/10.1101/2024.04.08.588518; this version posted April 9, 2024. The copyright holder for this preprint (which was not certified by peer review) is the author/funder, who has granted bioRxiv a license to display the preprint in perpetuity. It is made available under aCC-BY 4.0 International license.

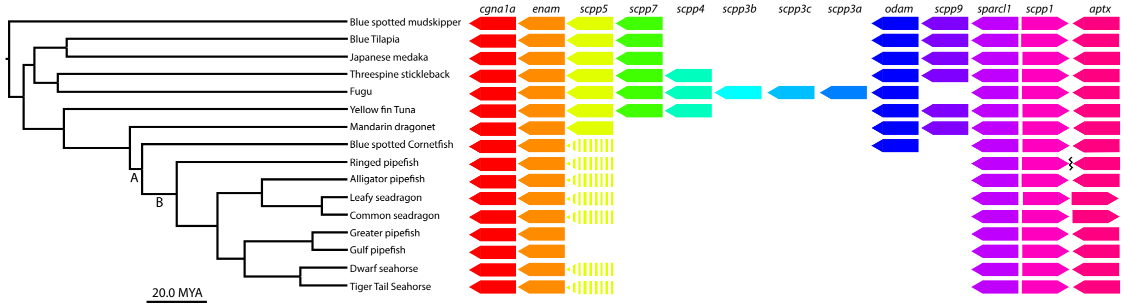

Figure S7: *Scpp gene cluster analysis identifies most Scpp losses are not unique to syngnathids.* A) denotes the Syngnathoidei lineage which contains syngnathids and blue spotted cornetfish (among other fish) to highlight that this clade contains various tooth alterations. Syngnathids lack teeth completely and cornetfish have small teeth limited to the lower jaw. B) denotes solely the syngnathid clade, which completely lacks teeth. The phylogenetic tree was time calibrated using Hughes et al. 2018 and Stiller et al. 2022.

bioRxiv preprint doi: https://doi.org/10.1101/2024.04.08.588518; this version posted April 9, 2024. The copyright holder for this preprint (which was not certified by peer review) is the author/funder, who has granted bioRxiv a license to display the preprint in perpetuity. It is made available under aCC-BY 4.0 International license.

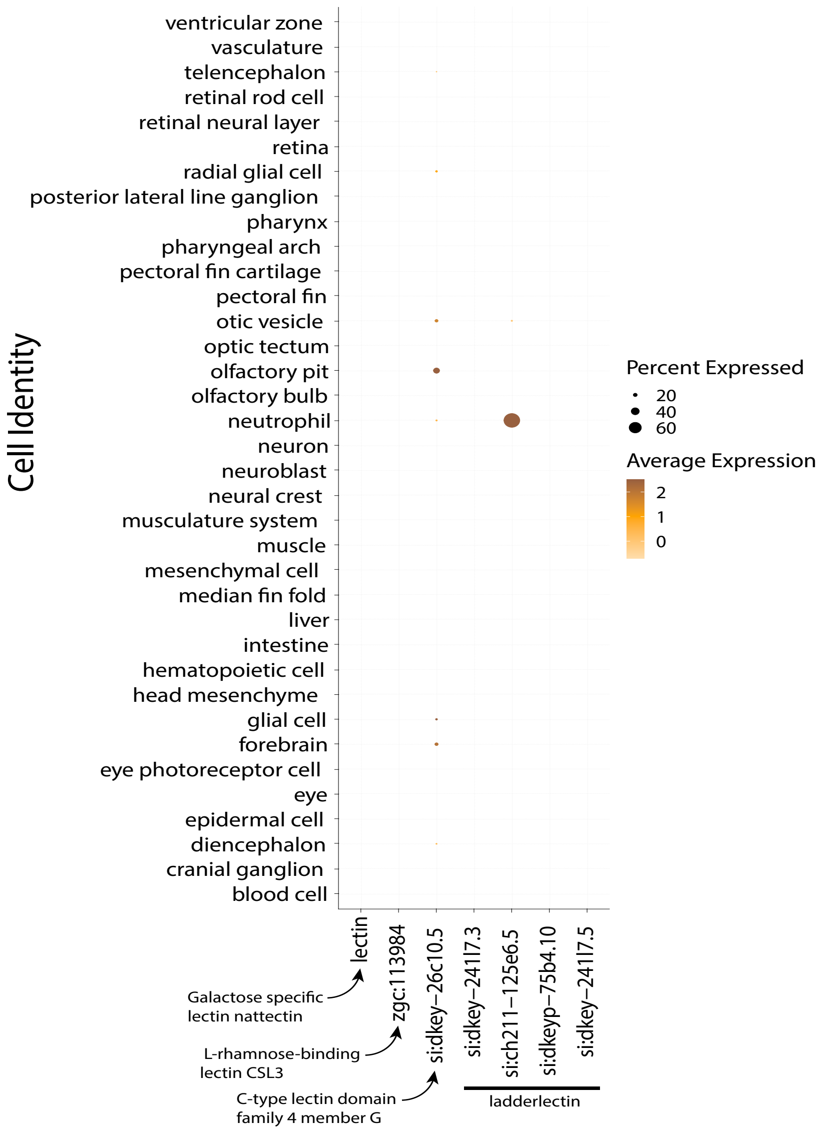

Figure S8: *3dpf Zebrafish do not express lectin genes in epidermal cells.* This dotplot shows the marker genes on the x-axis and y-axis, organized by cell type. The size of the dot represents the percentage of cells that express the gene in each cluster. The darkness of the dot represents the average expression of the genes.
